# The Monocarboxylate Transporter SLC16A6 Regulates Adult Length in Zebrafish and Is Associated With Height in Humans

**DOI:** 10.3389/fphys.2018.01936

**Published:** 2019-01-14

**Authors:** Santhosh Karanth, Amnon Schlegel

**Affiliations:** ^1^University of Utah Molecular Medicine Program, University of Utah School of Medicine, Salt Lake City, UT, United States; ^2^Division of Endocrinology, Metabolism & Diabetes, Department of Internal Medicine, University of Utah School of Medicine, Salt Lake City, UT, United States; ^3^Department of Nutrition and Integrative Physiology, College of Health, University of Utah, Salt Lake City, UT, United States; ^4^Department of Biochemistry, University of Utah School of Medicine, Salt Lake City, UT, United States

**Keywords:** height, SLC16A6, human, zebrafish, length

## Abstract

When fasted as larvae or fed ketogenic diets as adults, homozygous zebrafish *slc16a6a* mutants develop hepatic steatosis because their livers cannot export the major ketone body β-hydroxybutyrate, diverting liver-trapped ketogenic carbon atoms to triacylglycerol. Here, we find that *slc16a6a* mutants are longer than their wild-type siblings. This effect is largely not sexually dimorphic, nor is it affected by dietary fat content on a pure genetic background. A mixed genetic background alters the proportionality of mass to length modestly. We also observe that non-coding variations in the 5′-untranslated region and first intron, and coding variations within the fifth exon of the orthologous human gene locus *SLC16A6* are highly significantly associated with human height. Since both zebrafish and human orthologs of SLC16A6 are expressed in multiple locations, this gene likely regulates height through modulating transport of monocarboxylic acids in several tissues.

## Introduction

Previously, we isolated a zebrafish mutant with nutritionally suppressible hepatic steatosis, revealing that the liver has a dedicated β-hydroxybutyrate transporter required during fasting, Slc16a6a (Hugo et al., 2012). The molecular lesion within the encoding gene causes a complete loss of protein expression (hereafter *slc16a6^-^*^/^*^-^*). The hepatic steatosis phenotype in *slc16a6^-^*^/^*^-^* larvae could be rescued by forced expression of both the wild-type (WT) zebrafish *slc16a6a* and the orthologous human *SLC16A6* cDNAs in the livers of mutants.

In adult *slc16a6^-^*^/^*^-^* animals, we revealed a molecular mechanism for this selective diversion of carbon atoms to fatty acyl chains (and into triacylglycerol) but not into cholesterol (Karanth et al., 2013). By feeding isocaloric ketogenic diets (of both low and high fat composition) to *slc16a6^-^*^/^*^-^* animals, we caused massive hepatic steatosis to occur, with a lipid composition similar to that seen in fasted larvae (i.e., high triacylglycerol accumulation and low cholesterol accumulation). Furthermore, we detected an accumulation of polyunsaturated fatty acyl-Coenzyme A thioesters (PUFA-CoAs), activated intermediates of neutral and phospholipid synthesis. We demonstrated that PUFA-CoAs are competitive inhibitors of the rate limiting enzyme of cholesterol biosynthesis, 3-methyl-3-hydroxyglutaryl-CoA reductase (Hmgcr), providing *in vivo* confirmation of a previously long-standing *in vitro* observation (Karanth et al., 2013).

In our morphometric analyses of *slc16a6^-^*^/^*^-^* adults, we observed an increase in length and proportionate increase in mass. Here, we confirm this finding in a more detailed study showing that sex, dietary fat content, and (at least 2) genetic background(s) do not modulate this increase in length. Additionally, we find that in human population genetic studies, the *SLC16A6* locus is strongly associated with human height. We present 5′-untranslated region (UTR), first intronic, and missense fifth-exonic variations in the *SLC16A6* gene that are associated with human adult height. The SLC16A6 transporter may be a determinant of adult height.

## Materials and Methods

### Experimental Animals

This study was carried out in accordance with the recommendations of the Institutional Animal Care and Use Committee of the University of Utah. The protocol was approved the Institutional Animal Care and Use Committee of the University of Utah.

### Zebrafish

The *red moon^*s951*^* null allele of *slc16a6* was as we reported (Hugo et al., 2012). It was identified in a mixed-background, multiple-transgenic line used in a genetic screen (Anderson et al., 2009). This allele is carried on the WIK background (Rauch et al., 1997), having been subjected to over 10 back-crosses in our facility. Homozygous WT WIK siblings of *slc16a6^-^*^/^*^-^* animals were used as a comparators in one cohort of animals that were genotyped at 3 months post-fertilization and sorted into equal density housing tanks as we described previously (Karanth et al., 2013). In a second cohort, WIK background *slc16a6^-^*^/^*^-^* animals were crossed to AB and heterozygous progeny carrying the *slc16a6a* mutation were crossed to generate homozygous carriers and homozygous non-carrier siblings for study. This second cohort of animals were genotype, housed, and fed exactly as the first cohort, as we described previously (Karanth et al., 2013); animals are fed at 0900, 1400, and 1800 in our facility.

### Diets

Two isoproteic and isocaloric diets differing in lipid content were formulated and prepared exactly as described; 3-month post-fertilization adults were fed these diets for 45 days (Karanth et al., 2009, 2013).

### Morphometric Analysis and Body Composition

At the conclusion of the dietary study, animal length and mass were measured, and condition factor was calculated. Length is defined as the distance between the tip of the snout and the caudal peduncle, and was recorded using a Vernier caliper.

### Human Genetic Data

Human genetic data was retrieved from the open-access Accelerating Medicines Partnership (AMP) Type 2 Diabetes Knowledge Portal, http://www.type2diabetesgenetics.org/, and is based on GIANT Consortium data deposited from Locke et al. (2015).

### Statistics

Microsoft Excel 2016 and Sigmaplot 14.0 used for statistical analysis. The significance level was set at *P* < 0.05. The differences between the means were analyzed by two-tailed *t*-test. The number of animals examined per cohort is shown.

## Results

### Adult *slc16a6^-^*^/^*^-^* Zebrafish Are Proportionately Longer

**FIGURE 1:**
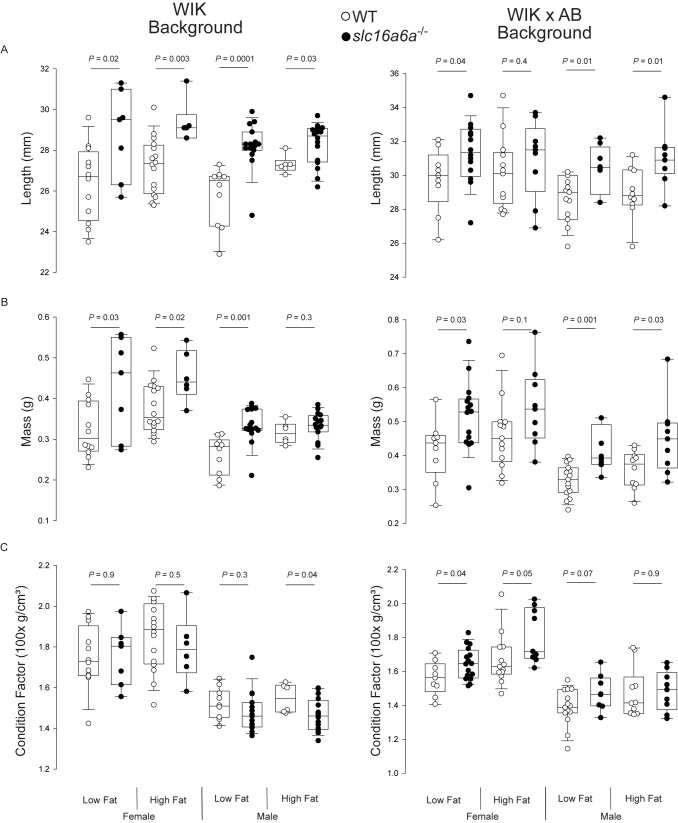
Adult *slc16a6^-^*^/^*^-^* animals are longer. Adult WT and *slc16a6^-^*^/^*^-^* animals (age 3 mpf; no difference in length or weight) of the indicated genetic backgrounds were fed low- and high-fat diets. After 45 days of feeding, length **(A)** and mass **(B)** were measured, and **(C)** condition factor was calculated. On both genetic backgrounds (WIK and WIK × AB), *slc16a6^-^*^/^*^-^* animals were longer and had higher mass, but had similar condition factors. Individual animal results are shown in tailed box plots marking median, 5, 25, 75, and 95th percentiles are shown.

We measured length and mass, and calculated the condition factor (Nash et al., 2006) of female and male WT and *slc16a6^-^*^/^*^-^* adults fed both low- and high-fat diets (Figures 1A–C). On both the WIK strain and a mixed WIK × AB backgrounds, *slc16a6^-^*^/^*^-^* animals of both sexes were longer and had higher masses, except for males on the WIK background, whose condition factors did not differ by genotype. The condition factor of females on the WIK background was not different, nor was the condition factor of males on the mixed genetic background fed high fat diets. The only additional factor that covaried was the effect of diet on WT WIK animals was diet (*P* = 0.013).

### Variations in the Human *SLC16A6* Gene Are Associated With Height

**FIGURE 2:**
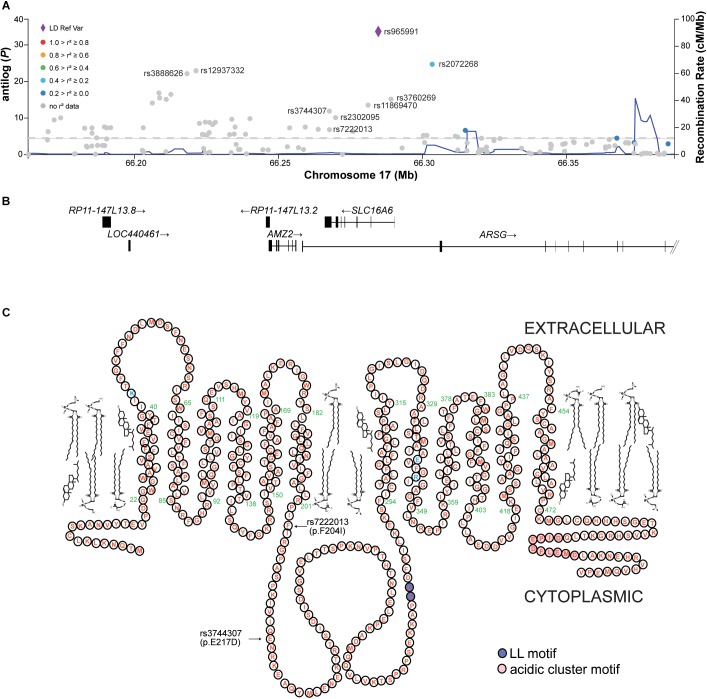
The human *SLC16A6* locus is associated with height. **(A)** In a cohort of over 300,000 human subjects, multiple variations in the 5′-UTR, first intron, and fifth exon of *SLC16A6* are significantly associated with height; there is a low rate of recombination across the *SLC16A6* gene. **(B)** The human *SLC16A6* gene is on the reverse strand of chromosome 17, with 2 non-coding initial exons followed by 5 coding exons. **(C)** Human SLC16A6 is an 8-pass plasma membrane protein, its catalytic residues are shown in blue and assigned these positions base on similarity to SLC16A1 (Manoharan et al., 2006). The dileucyl and acidic cluster motifs necessary for proper sorting are also indicated. The positions of the two missense variations associated with human height are within the large intracellular loop between the fourth and fifth transmembrane domains of this 12-pass transmembrane protein. The Glu^217^ residue is conserved residues among human, mouse and zebrafish orthologs; in mice and zebrafish the residues corresponding to Phe^204^ is an Ile residue (Hugo et al., 2012). Panels **(A,B)** from the open-access Accelerating Medicines Partnership (AMP) Type 2 Diabetes Knowledge Portal.

We were surprised to see that *slc16a6^-^*^/^*^-^* animals were longer in adulthood when fed ketogenic (i.e., inducing hepatic steatosis in the dietary study) diets, since these animals were faced with a metabolic challenge of being unable to export liver-derived ketone bodies (Hugo et al., 2012). Thus, we interrogated the GIANT UK Biobank genome-wide analysis (GWAS) for associations between the orthologous human gene *SLC16A6* and adult height (Locke et al., 2015). This multi-ethnic study included over 300,000 subjects. We observed multiple associations between single nucleotide variations within the 5′-UTR, first intron, and fifth codon of *SLC16A6* and adult height (Figures 2A,B). Linkage dysequilibrium information is not available for the variations identified (Figure 2B). The two coding variations associated with human height are mis-sense mutations in residues within the intracellular loop between transmembrane domains 4 and 5 of this plasma membrane-localized polytopic protein (Figure 2C).

## Discussion

Here, we find that the homozygous-viable zebrafish *slc16a6^-^*^/^*^-^* mutant strain, which develops steatosis either when fasting or when fed ketogenic diets, is proportionately longer than WT. In contrast, mass (and, hence, condition factor) is modified by genetic background and the fat content of the diet. These findings are paralleled by human population genetic observations that multiple variations (including two potentially deleterious coding mutations) within the orthologous human *SLC16A6* are associated with height, but not with body mass index (Locke et al., 2015). Our animal model data suggest that loss of SLC16A6 function promotes linear growth predominantly.

Since height is easily measured and does not change from young adulthood through middle age, it is an ideal trait for population genetics study of progressively sophisticated design (Guo et al., 2018). Large cohorts in which height and mass have been measured have identified hundreds of loci that contribute to these two continuous variables of human morphology (Locke et al., 2015; Marouli et al., 2017; Turcot et al., 2018). The molecular mechanisms for these associations are revealing new determinants of height, as well as confirming previously identified physiological mechanisms governing skeletal growth.

Since both zebrafish and human SLC16A6 orthologs are expressed widely, future studies will address where and possibly when this gene acts to limit vertical growth. While *slc16a6^-^*^/^*^-^* late larvae and early juveniles are sensitive to death by starvation (Hugo et al., 2012), the molecular lesion was found to be spontaneously present in the reference laboratory strain Tübingen (Howe et al., 2013). This inactivation likely confers more rapid growth in the aquarium, where food is deliberately abundant, and rapid physical and sexual maturation are under constant selection. Using cell- and developmental stage-limited rescue strategies, it may be feasible to establish when and where loss of *slc16a6a* expression drives increased linear growth. More generally, serial measurements of length and mass over a longer developmental window may reveal differences in growth rate; given the ease and cost of zebrafish husbandry, adequately powered and informative study with appropriate statistical analyses of covariates will be feasible (Tschop et al., 2012).

The available human population genetics data suggest that liver, brain, and endocrine pancreas are the primary sites of SLC16A6 expression in humans. While all three sites of SLC16A6 function might be important determinants of height, the association of loss-of-function coding variations within the pyruvate transporter *SLC16A11* gene and type 2 diabetes mellitus appears to be due to loss of SLC16A11 function in liver exclusively (Rusu et al., 2017). Indeed, the coding variants in SLC16A11 set a precedent for examining the roles of SLC16A6 F204I and E217D variants in altering substrate specificity, intracellular trafficking of the transporter, or both. Alternatively, since both of these substitutions are conservative, they might be proxies for causal variants that are nearby (Guo et al., 2018).

## Author Contributions

SK and AS designed the study, analyzed the data, wrote the manuscript, and performed the experiments. All authors discussed the results and commented on the manuscript.

## Conflict of Interest Statement

The authors declare that the research was conducted in the absence of any commercial or financial relationships that could be construed as a potential conflict of interest.
